# O.5.3-8 Prospects and challenges of real-world labs in physical activity promotion

**DOI:** 10.1093/eurpub/ckad133.259

**Published:** 2023-09-11

**Authors:** Maike Till, Peter Gelius, Karim Abu-Omar, Matthias Bergmann, Johanna Popp, Sven Messing

**Affiliations:** Friedrich-Alexander-Universität Erlangen-Nürnberg, Germany; Friedrich-Alexander-Universität Erlangen-Nürnberg, Germany; Friedrich-Alexander-Universität Erlangen-Nürnberg, Germany; Institute for social-ecological research Forschung, Frankfurt am Main, Germany; maike.till@fau.de; Friedrich-Alexander-Universität Erlangen-Nürnberg, Germany; Friedrich-Alexander-Universität Erlangen-Nürnberg, Germany

## Abstract

**Purpose:**

Participatory health promotion uses an array of different concepts to describe project processes and effects, including empowerment, capacity building, and capabilities. In practice, however, these concepts may hamper project scalability, compatibility with state-of-the-art health concepts, and efforts to counteract social inequities. We introduce the concept of real-world labs as a potential solution for current problems in participatory health promotion and investigate its practical applicability using the case example of an existing physical activity promotion project.

**Methods:**

We analyze current problems in participatory health promotion that emanate from the utilization of certain popular theoretical concepts. We then introduce real-world labs as a potential solution. Using the PArC-AVE-Project (Physical Activity-related Health Competence in Apprenticeship and Vocational Education) as a case example, we show how the approach can be used retrospectively to address the aforementioned issues.

**Results:**

Real-world labs are a type of transdisciplinary approach at the intersection of research and society. They help create an environment for flexibly testing innovative solutions under changing conditions using small-scale “real-world experiments” and feedback loops. However, the approach is so far not commonly used in participatory health promotion. The example of PArC-AVE illustrates that existing participatory projects can potentially be developed towards becoming real-world labs. Relevant perspectives for this include (a) the targeted integration of different actors into the participation process, (b) more variable approaches to scaling-up interventions, (c) a more flexible utilization of different participation methods, and (d) a better recognition of concepts such as planetary health.

**Conclusions:**

The real-world lab approach has the potential to increase the range of options available to participatory health and physical activity promotion. However, only further practical testing will show whether it is indeed effective in overcoming social inequities and ameliorating the high degree of context-dependence of many participatory projects.

